# How to ensure full vaccination? The association of institutional delivery and timely postnatal care with childhood vaccination in a cross-sectional study in rural Bihar, India

**DOI:** 10.1371/journal.pgph.0000411

**Published:** 2022-05-17

**Authors:** Mareike Schön, Esther Heesemann, Cara Ebert, Malavika Subramanyam, Sebastian Vollmer, Sebastian Horn

**Affiliations:** 1 Department of Paediatrics, University Medical Center Göttingen, Göttingen, Germany; 2 Center for Evaluation and Development, Mannheim, Germany; 3 RWI—Leibniz Institute for Economic Research, Essen, Germany; 4 Social Epidemiology, Indian Institute of Technology Gandhinagar, Palaj, Gandhinagar, Gujarat, India; 5 Chair of Development Economics, Center for Modern Indian Studies, University of Göttingen, Göttingen, Germany; 6 Department of Paediatrics, SRH Central Hospital Suhl, Suhl, Germany; University of Embu, KENYA

## Abstract

Incomplete and absent doses in routine childhood vaccinations are of major concern. Health systems in low- and middle-income countries (LMIC), in particular, often struggle to enable full vaccination of children, which affects their immunity against communicable diseases. Data on child vaccination cards from a cross-sectional primary survey with 1,967 households were used to assess the vaccination status. The association of timely postnatal care (PNC) and the place of delivery with any-dose (at least one dose of each vaccine) and full vaccination of children between 10-20 months in Bihar, India, was investigated. Bivariate and multivariable logistic regression models were used. The vaccines included targeted tuberculosis, hepatitis B, polio, diphtheria/pertussis/tetanus (DPT) and measles. Moreover predictors for perinatal health care uptake were analysed by multivariable logistic regression. Of the 1,011 children with card verification, 47.9% were fully vaccinated. Timely PNC was positively associated with full vaccination (adjusted odds ratio (aOR) 1.48, 95% confidence interval (CI) 1.06-2.08) and with the administration of at least one dose (any-dose) of polio vaccine (aOR 3.37 95% CI 1.79-6.36), hepatitis B/pentavalent vaccine (aOR 2.11 95% CI 1.24-3.59), and DPT/pentavalent vaccine (aOR 2.29 95% CI 1.35-3.88). Additionally, delivery in a public health care facility was positively associated with at least one dose of hepatitis B/pentavalent vaccine administration (aOR 4.86 95% CI 2.97-7.95). Predictors for timely PNC were institutional delivery (public and private) (aOR 2.7 95% CI 1.96-3.72, aOR 2.38 95% CI 1.56-3.64), at least one ANC visit (aOR 1.59 95% CI 1.18-2.15), wealth quintile (Middle aOR 1.57 95% CI 1.02-2.41, Richer aOR 1.51 95% CI 1.01-2.25, Richest aOR 2.06 95% CI 1.28-3.31) and household size (aOR 0.95 95% CI 0.92-0.99). The findings indicate a correlation between childhood vaccination and timely postnatal care. Further, delivery in a public facility correlates with the administration of at least one dose of hepatitis B vaccine and thus impedes zero-dose vaccination. Increasing uptake of timely PNC, encouraging institutional delivery, and improving vaccination services before discharge of health facilities may lead to improved vaccination rates among children.

## Introduction

Differences in childhood vaccination coverage notably contribute to the emergence of inequalities in child mortality and morbidity [1–3]. To achieve a proper immunization status, a minimum number of vaccination doses is necessary. Insufficient vaccination doses lead to the presence of a low immune response and poses uncertainty for specific immunities [4]. To assure herd-immunity and disrupt the transmission of vaccine preventable diseases (VPD) the population vaccination coverage needs to be high [5]. Under-vaccination and missing vaccinations remain a common public health problem. In 2019, 19.7 million children under the age of one, did not receive any or an incomplete number of routine vaccinations. Most of those children reside in low- and middle-income countries (LMIC) [5, 6]. In India, where this study is located, approximately 38% of children have not completed their vaccination schedule [7].

This study investigates perinatal services as potential determinants of children’s vaccination status in the North Eastern Indian state of Bihar. It explores the association of children’s vaccination with health care services (HCS) accessed at and after delivery, namely the place of delivery and timely postnatal care (PNC). These two indicators represent opportunities to encourage parents to complete their children’s vaccination schedule. Because individual vaccines are likely to differ in supply, vaccination rates, and number of required doses, both full vaccination status and any vaccination uptake of individual vaccines are investigated. In addition, predicting factors for institutional delivery and timely PNC attendance are studied to better understand the usage patterns of healthcare services.

Globally known associates of children’s full vaccination are child sex, birth order, resident area (urban/rural), parental education and literacy, caste (specific to India), religion, socio-economic status and access to health facilities [8–10]. Further, the lack of antenatal (ANC) and postnatal care as well as home delivery is associated with incomplete vaccination of children [8, 11–17].

The WHO defines postnatal care as care given to newborns and mothers immediately after birth and during the first 42 days of life and recommends at least four PNC visits, one of them within 24 hours after birth [18, 19]. Management of infections and timely vaccination are an essential part of proper PNC [20]. Further, delayed early vaccination was found to be associated with under-vaccination [21, 22]. This suggests that the provision of early perinatal services like timely PNC is associated with vaccination coverage. In India, districts with low ANC and PNC rates as well as lower rates of skilled birth attendance were found to have lower rates of full vaccination [8, 13, 14].

The state of Bihar reports particularly low vaccination rates which lie below the national average. In 2016, less than two-thirds (62%) of all children between 12–23 months received full vaccination against all six major preventable diseases (tuberculosis, pertussis, diphtheria, tetanus, polio and measles), despite the availability of these vaccines free of charge for all children [7].

Moreover, only 64% of mothers who gave birth during the last five years had a PNC check within two days. PNC coverage is the highest for deliveries in private health facilities (81%), followed by deliveries in public health facilities (69%), and the lowest for home deliveries (37%) [23]. In Bihar, during the last five years about three quarters (76%) of births took place in a health care facility, with numbers rising steadily [23]. Given the crucial relevance of the birth setting, following care and the emerging trend for the use of HCS it is worth exploring possible HCS quality improvements and their association with subsequent vaccination outcomes.

Few studies investigated the association of perinatal services and vaccination outcomes [8, 11–14, 16, 17]. This study can add to the existing knowledge on the relationship of perinatal services with children’s vaccination rates based on novel data from Bihar, India.

## Materials and methods

### Ethics statement

The study was approved by the ethics committee of the University of Göttingen on October 26th 2016 and the Indian Institute of Technology Gandhinagar. Each participant signed a written informed consent before the start of the interview.

### Study setting

The study uses primary data from a survey of recent mothers in Madhepura district, Bihar state, India. The data was collected between November and December 2016 for an endline survey of a randomised controlled trial investigating the impact of a participatory learning and action approach program on health, nutrition, and sanitation outcomes [24]. The sample size was deduced from power calculations in order to ensure enough statistical power for the rigorous evaluation of the trial.

### Study design and participants

Out of Madhepura’s thirteen sub-districts (blocks), six were chosen and 68 from a total of 95 gram panchayats were randomly sampled. A gram panchayat is a cluster of villages that constitute a local government body’s jurisdiction. The 68 gram panchayats comprised 180 villages. 40 villages, in which lists of pregnant women were missing, were excluded. In the remaining 140 selected villages, 1,967 households which were listed in a pregnancy register in 2015 (in local mother-child-centers: Anganwadi centers) were surveyed. The number of households sampled, per village, ranged from 5 to 49, depending on the village size. In 2016, 1,612 households with a child born recently, between 10 and 20 months of age, (average of 16 months) were revisited. The child had to live in the surveyed household to be included for this study. Exclusion criteria were death of the child and uncompleted pregnancy (miscarriage, abort, still birth). 166 children had died by 2016. Most of the attrition was caused by families having migrated or not being at home at the time of the survey. Of the remaining, 1442 participants were able to provide any type of information about children’s vaccination status and the questions of all included covariates. Of those, 1,011 households were able to present vaccination cards and were therefore eligible for the main analysis.

### Inclusivity in global research

Additional information regarding the ethical, cultural, and scientific considerations specific to inclusivity in global research is included in the S2 File.

### Study variables

Table 1 summarizes the national immunization schedule in India. The first dose of tuberculosis (BCG), hepatitis B, and polio vaccine are administered at birth and are followed up with the first dose of DPT vaccine at the age of six weeks. BCG is a single dose vaccine. Hepatitis B and polio require three vaccination doses with a spacing of four weeks. In January 2015, the pentavalent vaccine (haemophilus influenzae type b, DPT, hepatitis B) was introduced in Bihar. However, most children in the sample still received separate doses of hepatitis B and DPT vaccines. At nine to twelve months of age, single dose—measles and Japanese encephalitis—vaccine are administered. The outcomes of the analysis—children’s full vaccination status and any-dose vaccination—were defined based on WHO recommendations for the minimum number of vaccination doses [25] and the Indian national vaccination schedule:

1. **Fully vaccinated**: takes the value “1” if the child received one dose of BCG vaccine, at least three doses of polio vaccine, at least three doses of hepatitis B vaccine, at least three doses of DPT vaccine, and at least one dose of measles vaccine [26]. If the child missed one or more vaccine doses, the outcome takes the value of “0”. One or more pentavalent vaccine doses account for an equivalent number of hepatitis B and DPT vaccination doses.
2. **Any dose**: was defined individually for each vaccine against the following diseases: tuberculosis (BCG), polio, hepatitis B, DPT and measles. It takes the value of “1” if the child received at least one vaccination dose against the respective disease and takes the value of “0” if no dose was received.

**Table 1:** National immunization schedule, 2016 state recommendations for Bihar.

Age	Vaccine
Birth	Bacille Calmette-Guerin (BCG—tuberculosis), hepatitis B, oral polio vaccine (OPV)
6 weeks	OPV, pentavalent* (diphtheria, tetanus toxoid, pertussis, haemophilus influenza, hepatitis B), inactivated polio vaccine (IPV)**
10 weeks	OPV, pentavalent*
14 weeks	OPV, pentavalent*, IPV**
9–12 months	measles, Japanese encephalitis (JE)

In Bihar pneumococcal conjugate vaccine (PCV) has been launched in May 2017.

*Pentavalent vaccine was introduced in Bihar during January 2015.

**IPV was introduced across the country by April 2016.

The two explanatory variables of interest are place of delivery and timely PNC. Place of delivery was coded into three mutually exclusive dummy variables for home delivery, delivery in a private institution and delivery in a public institution based on self-reports of mothers. Timely PNC was defined as having received a postnatal check-up within 24 hours by a doctor, Auxiliary Nurse Midwife (ANM) or General Nurse Midwife (GNM).

All estimations controlled for characteristics of the mother, socio-cultural and economic household characteristics, and characteristics of children. Mothers’ characteristics were maternal age (14–23 years, 24–35 years, > 35 years), education level (no schooling, primary school, secondary school or higher), a dummy variable assessing if the mother was involved in the decision making regarding her child’s health care (maternal involvement) and membership in a self-help group. All determinants—except the latter—are known to be associated with childhood vaccination [8, 27–29] and to be correlated with the place of delivery and PNC [30–35]. Membership in a self-help group was included due to the survey design. Household variables included were household size, health insurance, the religion of the household (Hindu, Non-Hindu), and household wealth. Although caste is of reasonable relevance it was not included in our model due to the lack of bivariate association with outcome variables in previous base data set analyses. Wealth quintiles were based on an index generated from principal component analysis of household assets and housing quality. Household amenities, durable goods, and assets were measured, scored, and subsequently classified into quintiles ranging from poorest to richest. Questions concerning electricity, toilet facility, household assets (chair, table, bed) and vehicles (bicycle, motorcycle, cart), livestock and land ownership, as well as ownership of electronic devices (radio, mobile phone, land line, refrigerator, watch, electric fan), type of fuel for cooking, roofing and flooring materials were included. Children’s characteristics included were sex, age (12 months and younger, > 12 months), and number of older siblings.

### Data source and measurement

All information was recorded by trained, local enumerators during structured interviews using electronic questionnaires with the mother of the child in each household. Information about specific vaccinations of the child for each recommended vaccine in the national immunization schedule was obtained from documented evidence by vaccination or health cards. The common Mother Child Protection card (MCP) established in Bihar allows the tracking of perinatal health and is available at all points in the heath system [36]. If the vaccination or health card was not available, responses from maternal recall were recorded.

### Bias

Sample selection bias may affect the external validity of the results. The survey only included registered pregnant women and the estimation sample was restricted to observations with vaccination records that were documented in a vaccination or health card. To test the extent of bias of the latter estimation sample restriction, a sensitivity analysis including all children, i.e. with documented vaccination evidence and vaccination evidence from maternal reports, was conducted (Tables E-G in S1 File).

### Data analysis

A cross-sectional study design to determine the association of perinatal HCS with full and any-dose vaccination was used. A bivariate model was fitted for each covariate and outcome variable. All variables with a p-value of less than 0.1 were included in a multinominal logistic regression model. To avoid multicollinearity among the explanatory variables, a Pearson’s R correlation statistic with a cutoff of r > 0.5 for all pair combinations of covariates was conducted (Table A in S1 File). Adjusted odds ratios (aOR) were estimated using multivariable models to identify the association of timely PNC and the place of delivery with full and any-dose vaccination. A p-value < 0.05 was considered as statistically significant. For each outcome, three models were estimated introducing all variables in a step wise fashion. In the first model, the primary explanatory variables were introduced (Table B in S1 File). In the second model, covariates were added (Table C in S1 File), and in the third model, fixed effects at the block (sub-districts/administrative unit) level were included to control for regional characteristics in the six different blocks (Table D in S1 File). Standard errors are clustered on panchayat level. In the sensitivity analysis, the estimation was conducted with the full sample and an indicator for the availability of a vaccination card was included as a covariate (Tables E and F in S1 File).

To better understand the drivers of timely PNC and institutional delivery, a multivariable logistic regression analysis was conducted using previous contact to the health system, socio-economic, maternal, and household characteristics as predictors. The same maternal and household-level variables were used as in the regression on the vaccination status and a dummy for belonging to a Schedule Caste, Scheduled Tribe, or so-called Other Backwards Caste was added. This analysis includes all children, irrespective of their vaccination evidence.

All analyses were conducted using the statistical software Stata^®^ 13 (StataCorp LLC, College Station TX, USA).

## Results

### Sample characteristics

The descriptive characteristics of the study population are presented in Table 2. About 85% of children belong to Hindu households. Approximately 21% of children belong to the privileged General Caste category, 79% either to historically disadvantaged Scheduled Castes, Scheduled Tribes or to so-called Other Backward Castes. Households were divided into five wealth quintiles. These wealth quintiles showed differences regarding the vaccination evidence, with the poorest quintile being the most prominent among participants with a lack of vaccination evidence (24.0%). About one fifth (21.7%) of all households had health insurance. Roughly three-quarters (74.5%) of the children’s mothers never went to school or did not complete primary school and 51% of mothers were between 24–35 years of age. Slightly more than half of the children surveyed were male (53%). Approximately two thirds (64%) of all mothers were involved in decision making concerning their children’s health care. Table 2 further shows that 53% of all mothers received ANC at least once. 68% of the children were born either in private or public health facilities. 47% of children and mothers received a check-up within 24 hours after birth.

**Table 2:** Descriptive statistics of households according to data availability.

	Card verification		Maternal recall		Overall	
Characteristics	N	%	N	%	N	%
**Received at least one ANC visit**	996	100.0	421	100.0	1,417	100.0
No	434	43.6	236	56.1	670	47.3
Yes	562	56.4	185	43.9	747	52.7
**Birth setting**	1,006	100.0	430	100.0	1,436	100.0
Institutional, public	622	61.8	261	60.7	883	61.5
Institutional, private	84	8.3	29	6.7	113	7.9
Home birth	300	29.8	140	32.6	440	30.6
**Timely PNC**	959	100.0	408	100.0	1,367	100.0
No	540	56.3	187	45.8	727	53.2
Yes	419	43.7	221	54.2	640	46.8
**Sex of child**	1,011	100.0	430	100.0	1,441	100.0
Female	489	48.4	189	44.0	678	47.1
Male	522	51.6	241	56.0	763	52.9
**Age of the child > 12 months**	1,011	100.0	431	100.0	1,442	100.0
No	26	2.6	19	4.4	45	3.1
Yes	985	97.4	412	95.6	1,397	96.9
**Number of older siblings**	957	100.0	378	100.0	1,335	100.0
No siblings	304	31.8	113	29.9	417	31.2
1–2 siblings	486	50.8	177	46.8	663	49.7
3 or more siblings	167	17.5	88	23.3	255	19.1
**Age of mother**	998	100.0	428	100.0	1,426	100.0
15–23 years	303	30.4	113	26.4	416	29.2
24–34 years	495	49.6	233	54.4	728	51.1
35 years and older	200	20.0	82	19.2	282	19.8
**Education of mother**	1,011	100.0	431	100.0	1,442	100.0
No schooling	746	73.8	328	76.1	1,074	74.5
Primary school	88	8.7	31	7.2	119	8.3
Middle school or higher	177	17.5	72	16.7	249	17.3
**Maternal involvement health care child**	1,009	100.0	429	100.0	1,438	100.0
No	364	36.1	153	35.7	517	36.0
Yes	645	63.9	276	64.3	921	64.0
**Self-help-group**	999	100.0	424	100.0	1,423	100.0
No	738	73.9	327	77.1	1,065	74.8
Yes	261	26.1	97	22.9	358	25.2
**Household size**	1,011	100.0	431	100.0	1,442	100.0
<4 members	52	5.1	20	4.6	72	5.0
4–7 members	463	45.8	191	44.3	654	45.4
>7 members	496	49.1	220	51.0	716	49.7
**Health insurance**	1,009	100.0	430	100.0	1,439	100.0
No	779	77.2	348	80.9	1,127	78.3
Yes	230	22.8	82	19.1	312	21.7
**Religion**	1,001	100.0	417	100.0	1,418	100.0
Non-Hindu	133	13.3	80	19.2	213	15.0
Hindu	868	86.7	337	80.8	1,205	85.0
**Wealth Quintile**	957	100.0	408	100.0	1,365	100.0
Poorest	174	18.2	98	24.0	272	19.9
Poorer	191	20.0	84	20.6	275	20.1
Middle	200	20.9	78	19.1	278	20.4
Richer	208	21.7	65	15.9	273	20.0
Richest	184	19.2	83	20.3	267	19.6
**Historic caste category**	1,008	100.0	431	100.0	1,439	100.0
General category	214	21.2	92	21.3	306	21.3
Other s.-c. backward class, scheduled caste or tribe	794	78.8	339	78.7	1,133	78.7
**Total**	1,011		431		1,442	

Only households with vaccination evidence in form of vaccination cards and with complete data were included in the analytic sample. Numbers may not add up due to missing observations. S.-c., so-called.

1,011 participants with vaccination cards were eligible for the main analysis. Several variables had some missing data (birth setting 0.49%, timely PNC 5.14%, ANC 1.48%, child’s sex 0%, age of the child 0%, age of the mother 1.98%, education of mother 1.48%, involvement mother in health care decision 0.20%, number of older siblings 5.34%, household size 0%, wealth quintile 5.43%, religion 0.99%, insurance 0.20%, self-help-group 1.19%). Observations with incomplete information in outcome and explanatory variables were removed prior to the analysis by listwise deletion in Stata. The final estimation sample comprised of 809 observations.

### Descriptive statistics for full vaccination and any-dose vaccination

Vaccination status of children according to the source of information and for the overall study population is shown in Table 3 for all relevant vaccines under investigation. Comparing the vaccination coverage from the vaccination card and from maternal recall shows a substantially lower vaccination rate for children with vaccination card verification, than without. The difference is particularly salient for hepatitis B and polio vaccines. This divergence might be driven by the quantities of vaccines required, which challenge an accurate recall. In the following analysis, we therefore focus on the study sample with card verification.

**Table 3:** Vaccination status according to data availability by vaccination documentation.

	Card verification				Maternal recall				Overall			
	Full		Any		Full		Any		Full		Any	
Characteristics	N	%	N	%	N	%	N	%	N	%	N	%
All	484	47.9	1004	99.3	253	64.4	413	96.9	737	52.5	1417	98.6
BCG vaccine	987	97.6			363	93.1			1,350	96.4		
Polio vaccine	807	79.8	926	91.6	323	87.3	371	96.1	1,130	81.8	1,297	92.8
Hepatitis B or pentavalent vaccine	552	54.6	855	84.6	307	81.6	364	94.8	859	61.9	1,219	87.4
DPT or pentavalent vaccine	728	72.0	852	84.3	284	73.6	352	94.1	1,012	72.4	1,204	86.9
Measles vaccine	880	87.0			308	86.0			1,188	86.8		
Total	1,011	100	1,011	100	431	100	431	100	1,442	100	1,442	100

Only households with vaccination evidence in form of vaccination cards and with complete data were included in the analytic sample. Full: Full vaccination status versus no or any vaccination, Any: Any vaccination dose versus no vaccination dose of the respective vaccine. BCG and measles only require one vaccination dose to be fully vaccinated against the disease. For the other vaccines three vaccination doses were considered fully vaccinated.

**Fig 1:**
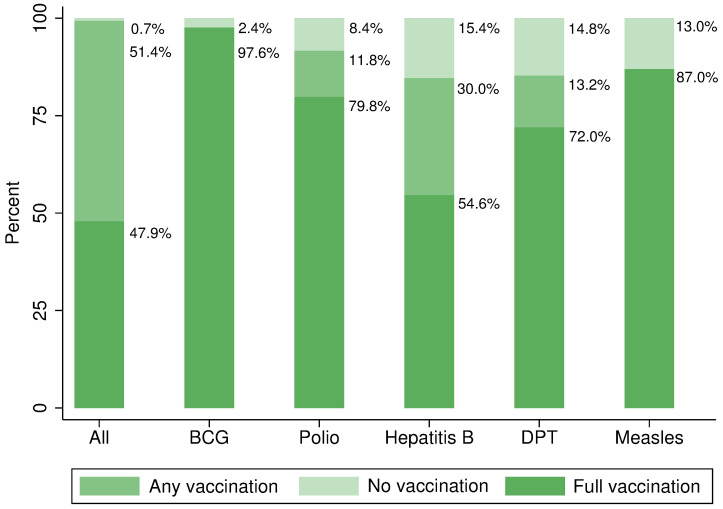
Vaccination status by vaccine. Graph refers to analytical sample; respondents with card verification and complete data. “No vaccination” is equivalent to missing vaccination for the respective vaccine. “All” presents a summary over all vaccinations recommended until the age of twelve months by the WHO and the national vaccination schedule of India; one dose of BCG vaccine, three doses of polio vaccine, three doses of hepatitis B vaccine and three doses of DPT vaccine and one dose of measles vaccine. Pentavalent vaccine doses account for the equivalent number of hepatitis B and DPT vaccination doses.

The vaccination status breakdown by vaccine for the main analytical sample is depicted in Fig 1. The graph distinguishes between fully vaccinated, any-dose (but not all) and not vaccinated. Looking at vaccines separately, BCG showed the highest vaccination coverage with 97.6%. The lowest coverage for full vaccination was found for the hepatitis B vaccine with 54.6%. However, a comparably large proportion of children was partially vaccinated against hepatitis B (30%). 87% of all children received at least one measles vaccine dose. Half of all surveyed children were fully vaccinated. Less than 1% of the children did not receive any vaccination at all.

### Associations of vaccination coverage with place of delivery and timely PNC

Bivariate statistics showed that the historic caste category and wealth quintile were not associated with full or any-dose vaccination status. All other control variables (ANC, sex and age of the child, number of older siblings, mother‘s age and education, maternal involvement, self-help-group membership, household size, health insurance and religion) showed a significant association with full or any-dose vaccination and were introduced to the multivariable logistic regression model. Wealth showed significant association with perinatal health care uptake (Table 5) and was thus included in the multivariable regression model. Pearson’s R correlation statistic did not show collinearity among the included variables (Table A in S1 File).

Results of the logistic regression model are displayed in Table 4. It shows results based on a model with all covariates clustering at the panchayat level. Timely PNC was statistically significantly and positively associated with children’s full vaccination status: children who received timely PNC had higher odds (aOR 1.48, 95% CI 1.06–2.08) of being fully vaccinated. Further, children with timely PNC had higher odds of receiving any dose of vaccine plans with multiple doses, namely polio (aOR 3.37 95% CI 1.79–6.36), hepatitis B (aOR 2.11 95% CI 1.24–3.59) and DPT vaccine (aOR 2.29 95% CI 1.35–3.88). There was no statistically significant association between timely PNC and BCG or measles vaccination. The estimation results showed no association between the place of delivery and full vaccination status. However, children born in a public facility had higher odds of being vaccinated at least once against hepatitis B (aOR 4.86, 95% CI 2.97–7.95).

**Table 4:** Association of perinatal care (birth setting and timely PNC) with the vaccination status of children aged 10–20 months in Bihar.

	Full vaccination	Any vaccination				
Predicting variables	All	BCG	Polio	Hepatitis B	DPT	Measles
**Birth setting** (Ref: Home birth)						
Institutional, public	1.07	1.97	1.59	**4.86** ***	1.36	1.03
	[0.74,1.55]	[0.69,5.64]	[0.95,2.66]	[2.97,7.95]	[0.89,2.07]	[0.59,1.77]
Institutional, private	1.55	1.05	0.52	1.51	0.78	1.77
	[0.82,2.93]	[0.26,4.23]	[0.20,1.34]	[0.71,3.22]	[0.37,1.64]	[0.41,7.60]
**Extra care**						
Timely PNC	**1.48** *	1.93	**3.37** ***	**2.11** **	**2.29** **	0.94
	[1.06,2.08]	[0.82,4.53]	[1.79,6.36]	[1.24,3.59]	[1.35,3.88]	[0.58,1.52]
Observations	809	809	809	809	809	809
Prob >*chi*^2^	0.00	0.00	0.01	0.00	0.00	0.33

Boldface stars indicate statistical significance:

* (p<0.05),

** (p<0.01),

*** (p<0.001).

Logistic regression results are presented as OR with confidence interval in brackets. The analytical sample includes all respondents with card verification and complete data. For consistency across models, all models adjust for ANC, sex and age of the child, number of older siblings, mother‘s age and education, maternal involvement in the health care decisions regarding her child, membership of a self-help-group, household size, health insurance and religion. The model with introduced fixed effects controlling for block characteristics is shown in the Table D in S1 File. Results are similar to the shown table. Odds of having received at least a single vaccination for BCG, polio, hepatitis B or DPT vaccination are compared by birth setting and the provision of timely PNC. Further odds of having received full vaccination for all recommended vaccines (All) are compared by birth setting and the provision of timely PNC. Ref, Reference; OR, Odds ratio; BCG, Bacille Calmette-Guerin; DPT, diphtheria/pertussis/tetanus.

Unadjusted estimates, estimates including block dummies and results for the full sample of children with vaccination evidence from maternal recall or the vaccination card can be found in Tables B and G in S1 File. The sensitivity analysis confirmed the results of the main analyses.

Several covariates showed associations with the outcome variables (Table C in S1 File). Children of mothers involved in health care decision making (aOR 2.19, 95% CI 1.47–3.25), boys (aOR 1.30, 95% CI 1.03–1.63), and children in Hindu households (aOR 1.90, 95% CI 1.05–3.44) had significantly higher odds of being fully vaccinated. Similar associations were found for hepatitis B and DPT vaccination, respectively. The odds of being fully vaccinated or having received any BCG (aOR 1.09 95% CI 1.01–1.19), polio (aOR 1.11 95% CI 1.02–1.21), hepatitis B (aOR 1.11 95% CI 1.05–1.17) or DPT (aOR 1.10 95% CI 1.02–1.18) vaccines increased for children with each additional household member. The odds of having received at least one dose of measles vaccine were higher for children of better educated women (aOR 2.54 95% CI 1.20–5.37) and of mothers who had attended ANC at least once (aOR 1.73 95% CI 1.03–2.92).

The associations of previous health care contacts, socio-economic, maternal and household characteristics with the place of delivery and timely PNC uptake are subsequently shown in Table 5. Contact to health care providers during pregnancy in form of at least one ANC visit was significantly positively associated with timely PNC (aOR 1.59 95% CI 1.18–2.15) and institutional delivery in private (aOR 2.38 95% CI 1.56–3.64) and public facilities (aOR 2.70 95% CI 1.96–3.72). Institutional birth was significantly positively and strongly associated with timely PNC (aOR 2.38 95% CI 1.56–3.64 for public facilities, aOR 2.70 95% CI 1.96–3.72 for private facilities). Children from wealthier and smaller families had higher odds to receive timely PNC. Delivery in a private facility was significantly positively associated with the two uppermost wealth quintiles (aOR 4.99 95% CI 1.80–13.82 and aOR 6.06 95% CI 2.25–16.30) and living in a Hindu household (aOR 2.21., 95% CI 1.14–4.28). Wealth, mother’s education, and being of small family size were insignificantly positively associated with deliveries in public institutions. Maternal age, maternal involvement, self-help group membership, belonging to a less disadvantaged caste, and the number of older siblings were not associated with the place of delivery or timely PNC.

**Table 5:** Predictors of perinatal health care uptake.

		Birth setting	
	Timely PNC	Institutional, public	Institutional, private
**Birth setting** (Ref: Home birth)			
Institutional, public	**2.70** ***		
	[1.96,3.72]		
Institutional, private	**2.38** ***		
	[1.56,3.64]		
**Extra care**			
Received at least one ANC visit	**1.59** **	**2.18** ***	**3.71** ***
	[1.18,2.15]	[1.73,2.74]	[2.44,5.63]
**Maternal characteristics**			
***Age** (Ref: < 24 years)*			
24–34 years	0.98	0.82	0.87
	[0.73,1.33]	[0.59,1.14]	[0.45,1.67]
35 years or older	0.94	0.93	1.35
	[0.63,1.39]	[0.60,1.43]	[0.65,2.79]
***Education** (Ref: No schooling)*			
Primary school	0.71	1.17	1.12
	[0.43,1.17]	[0.68,1.99]	[0.41,3.02]
Middle school or higher	0.87	1.69	1.83
	[0.62,1.22]	[0.99,2.87]	[0.88,3.82]
Maternal involvement health care child	1.10	0.95	0.94
	[0.86,1.41]	[0.71,1.28]	[0.52,1.71]
Self-help-group	1.21	0.95	0.90
	[0.86,1.71]	[0.71,1.27]	[0.50,1.63]
**Household characteristics**			
Household size	**0.95** *	0.97	0.99
	[0.92,0.99]	[0.91,1.03]	[0.91,1.08]
Health insurance	0.80	0.86	0.74
	[0.56,1.16]	[0.63,1.16]	[0.40,1.37]
Hindu	0.72	1.23	**2.21** *
	[0.47,1.11]	[0.78,1.95]	[1.14,4.28]
***Wealth quintile** (Ref: Poorest)*			
Poorer	1.34	1.08	2.23
	[0.87,2.05]	[0.64,1.82]	[0.74,6.70]
Middle	**1.57** *	1.24	1.65
	[1.02,2.41]	[0.78,1.97]	[0.63,4.31]
Richer	**1.51** *	1.52	**4.99** **
	[1.01,2.25]	[0.96,2.40]	[1.80,13.82]
Richest	**2.06** **	1.30	**6.06** ***
	[1.28,3.31]	[0.81,2.09]	[2.25,16.30]
General Caste category or other	1.43	0.91	0.86
	[1.00,2.05]	[0.62,1.34]	[0.44,1.71]
Number of older siblings	0.97	0.91	0.86
	[0.85,1.10]	[0.82,1.01]	[0.66,1.13]
Observations	1110	1020	422
Prob >*chi*^2^	0.00	0.00	0.00

Boldface stars indicate statistical significance:

* (p<0.05),

** (p<0.01),

*** (p<0.001).

Logistic regression results are presented as OR with confidence interval in brackets. Standard errors clustered on panchayat level. Column 2 compares children delivered in public facilities and home births, children born in private facilities are excluded. Column 3 compares children delivered in private facilities and home births, children born in public facilities are excluded. Omitted categories are: Maternal age below 24 years, no schooling or incomplete primary school, poorest wealth quintile. Ref, Reference.

## Discussion

Community-based data from 809 participants living in rural Bihar state in India was used to examine the associations of institutional birth and timely PNC with full and any-dose vaccination. The results showed that timely PNC was significantly positively associated with children’s vaccination status. This contributes to the evidence on the association of PNC and children’s vaccination status and to our knowledge, is the first study to specifically examine the association of timely PNC.

The vaccination coverage for BCG (96.4%) was very high, suggesting a broad level of HCS access. The low levels of literacy, as well as the distributions of age, religion, and caste were in line with district wide statistics [7, 37]. The proportion of mothers who received timely PNC (46.8%) was comparable to the district statistics (urban 52.6%, rural 41.1%) [7].

### Vaccination coverage

The full vaccination coverage in the study sample (52.5%) was lower than estimates for Bihar based on the National Family and Health Survey in 2015/2016 (61.7%). The vaccination rates of BCG and polio were slightly higher in the study sample than in the aforementioned survey and lower for hepatitis B and DPT (BCG 91.6%, polio 72.9%, hepatitis B 65.5%, DPT 80.1%) [7]. Because the pentavalent vaccine was introduced in January 2015, the supply chain may not have been set up completely at the time of the survey and supply shortages may have caused low rates of DPT and hepatitis B vaccination [38]. This might also be the reason for DPT and hepatitis B vaccine showing the largest discrepancies between card verification and maternal recall in coverage estimates.

### Birth setting

Children born in a public or private facility were not more likely to have been fully vaccinated than children born at home. However, the odds of receiving at least one dose of hepatitis B were significantly increased when birth took place in a public facility in comparison to home deliveries. The distinct result for hepatitis B might be due to the novel introduction of hepatitis B to the Indian Universal Immunization Program (UIP). It was introduced in 2011 to the Indian UIP in Bihar and at the time of the survey lower rates compared to other vaccines were commonly observed [38, 39]. The main reasons for the low hepatitis B coverage were found to be poor stock management, perceived high costs, fear of wastage of the vaccine, and insufficient knowledge about the vaccination schedule among health workers [40].

Previous evidence suggests that public facilities possibly perform better than private facilities in the administration of vaccines [8]. This is in line with the presented data for hepatitis B vaccine showing that in opposition to delivery in public facilities, delivery in a private facility did not protect from zero-dose vaccination compared to home deliveries. Reasons for this might be the missing governmental mandate to ensure vaccination for the poor in private facilities. Policies or financial incentives for adequate vaccination practices are not present. Supply in public facilities, i.e. for hepatitis B or pentavalent vaccine might be better than in private facilities [41].

Other studies showed that institutional delivery is positively associated with full vaccination [12, 15, 42]. In this study a strong positive and significant association between institutional delivery and vaccination status was not observable. Yet, none of the mentioned studies controlled for timely PNC. This implies that timely PNC might be an essential pillar of adequate service delivery in health care facilities directly after birth.

The choice of place of delivery was seen to be highly correlated with initial contact to health workers through ANC and household wealth. These findings are in line with studies from Nepal, Bangladesh, Pakistan and other parts of India [43–46]. Hence, although there was no direct association between ANC and vaccination status, there potentially could be an indirect association through institutional delivery. It underscores the importance of early contact of health workers with pregnant women, especially those coming from a low-income background.

### Postnatal care

Timely PNC showed a positive and significant association with full vaccination status and a significant positive association with vaccination against individual diseases which require multiple doses of vaccine—polio, hepatitis B and DPT. There was no association with any-dose of BCG or measles vaccination. The lack of association with BCG might be due to the overall high coverage rate of close to 100%.

The results add to the scarce literature on the association between vaccination coverage and PNC [8, 11, 13, 14, 16, 17]. Vaccinations are an essential component of timely PNC and PNC visits offer an opportunity to parents to learn about vaccination. Research showed that most women in Bihar who had their children vaccinated were motivated and supported by health workers. Mothers are often seen to rely on information given to them during their commonly sparse interactions with health care facilitators [47]. During PNC visits, health workers share information regarding the benefits, relevance and the schedule of routine vaccinations. Greater awareness of the child’s health issues among mothers who were provided with (ANC and) PNC was documented [20, 42]. Moreover, PNC visits might offer an opportunity for counselling on uncertainties concerning vaccinations. Motivation and information provided in PNC meetings possibly strengthens follow-up in the vaccination schedule.

This would suggest that not only the mere health care facility availability but also quality of service delivery, like timely PNC, matter for uptake and long-term motivation regarding vaccination. Prior research in low- and high-income countries found that delayed early vaccination is associated with under-vaccination [21, 22, 48]. Our findings suggest that timely PNC could be a reasonable intervention, in the form of a systematic program, to target early vaccination in order to improve full protective vaccination coverage. Implementing structured standardised timely PNC visits might be an effective and efficient measure for HCS quality improvement. Considering that facilitated birth tripled during the last ten years in Bihar, leveraging it to improve routine care in health care facilities could substantially help increase vaccination coverage rates [23].

Further analyses are needed to investigate differences in vaccination service delivery by provider and how they contribute to the heterogeneity in vaccination rates as well as the quality of PNC in health care facilities and during home visits [49].

Given the crucial role that timely PNC plays for complete childhood vaccination, it is important to note that the odds of receiving timely PNC increase not only with previous contact to the health system in form of ANC or institutional delivery, but also with household wealth, thus confirming findings from the previous study region [45, 46, 50–52]. Children growing up in poor households are less likely to be protected against childhood communicable diseases caused morbidity and mortality adding to their overall disadvantage in life.

### Confounding variables

The results of the confounding variables showing association with the vaccination status are well in line with existing evidence. Beliefs shape vaccination behaviour of parents and religion is highly predictive for children’s vaccination status. More specifically, children from Hindu households show higher odds for a full vaccination status [8, 9]. Moreover inequity in childhood vaccinations by sex was found all over India, with most prominent imbalances in Bihar [9]. Those can be explained by strong preferences for sons over daughters in the study area [53]. Further past studies have shown that resource allocation to children is strongly linked to decision making power of women in the household [54]. Whereas the effect on the nutritional status of the child is a global phenomenon, the effect on the vaccination status was primarily found in South Asia [29]. Our study confirms this finding once more by showing a strong correlation of female empowerment and completed vaccination schedule.

Additionally wealth is a known predictor for vaccination status, children with low socioeconomic status are less likely to be fully vaccinated [9]. Often low quality antenatal, delivery and postnatal care is primarily provided to people from less wealthy households [30, 32, 35, 55]. In conjunction this may lead to impaired provision of vaccination evidence.

### Limitations

Following limitations need to be noted. First, only pregnant women registered at an Anganwadi center were included in the study, which potentially affected the representativeness of the population at risk and may have led to a sample selection bias. According to the National Family and Health Survey in 2015/2016, around 70% of Bihari women hold a mother-child health card, indicating registration at public health facilities [7]. Our sample therefore excludes families without access to the public health system, or without motivation or knowledge on the benefits of a registered pregnancy. Second, about 20% of the initial sample was lost in the follow-up survey. While temporary absence of key household members and migration is probably unrelated to health care behaviour, child survival is probably positively linked to both PNC, institutional birth and vaccination of children. This survival bias might hence cause our results to be a lower bound estimate of the true relationship between place of delivery, PNC and vaccination status. Third, the main analysis only covers children with vaccination evidence based on vaccination cards. Mothers self-reporting bears the risk of socially desired reporting and difficulty of remembering specific vaccines. The validity of this method is uncertain [56]. For this reason, we only use recall vaccination information as a robustness check. This skews the sample slightly to the more affluent, non Hindu households as Table 2 shows. Sensitivity analysis by including children with mothers’ self-reported vaccination evidence in the sample though confirmed the main results of the restricted sample. However the lack of a ‘gold standard’ for the recording of the vaccine status is a reoccurring challenge affecting coverage estimates in LMIC. Last, given the nature of a cross-sectional study design we are unable to draw conclusions about the causal relationship between the place of delivery and timely PNC with children’s vaccination status.

The study shows the difficulty of capturing accurate vaccination information from households, when no administrative data or other official records are available. Even though card verification, which documents the date of each vaccine reliably is a cost efficient and easy tool to keep track of children’s vaccination status, the cards are made of paper and tend to wear. Providing them of more robust material might help the survival of the card through multiple years. The option to use digital records, instead of the print out card could also help to keep vaccination information available. Multiple rounds of data collection to ensure more accurate recall, collaboration with local health facilities or vaccination teams could be a possibility to improve data quality. Data imputation is a further method which could be considered when dealing with vaccination estimates [56].

## Conclusion

This cross-sectional study provides evidence for a positive association of institutional birth and timely PNC with vaccination coverage of infants and children. These findings suggest that health care system quality improvements with respect to vaccination coverage should consider timely PNC at initial health care provider contacts. Timely PNC visits can be a relevant measures for sharing vaccination information and motivate take up. Institutional health care providers should offer the opportunity for early postnatal service for women of all socio-economic groups and follow up on its attendance. Considering confounding factors, district health offices should stress the importance to vaccinate all infants independently off their sex or religious background at institutional health care providers. Differences in quality of PNC services and their effect on parents’ motivation, particularly in the case of non-vaccination, require further investigation.

## Supporting information

S1 Data Stata file comprising the minimal data set underlying analyses and figures. (DTA)

S1 File Tables A–H. (PDF)

S2 File Inclusivity in global research questionnaire. (DOCX)
