# Supplementary material for: How to ensure full vaccination? The association of institutional delivery and timely postnatal care with childhood vaccination in a cross-sectional study in rural Bihar, India
Source: PLOS Glob Public Health. 2022 May 17;2(5):e0000411. doi: 10.1371/journal.pgph.0000411 (PMC10021874; doi:10.1371/journal.pgph.0000411)
Supplement: S1 File — (PDF) [file pgph.0000411.s002.pdf]

S1 Tables

Table A. Collinearity among the predicting variable for the analytical sample.

		1	2	3	4	5	6	7	8	9	10	11	12	13	14
Institution of delivery	**1**	1.00													
At least one ANC visit	**2**	-0.17	1.00												
Timely PNC	**3**	-0.24	0.22	1.00											
Sex of the child	**4**	-0.02	0.04	0.02	1.00										
Age of child	**5**	-0.03	-0.03	-0.04	-0.07	1.00									
Age of mother	**6**	0.04	-0.03	-0.03	0.01	-0.06	1.00								
Education of mother	**7**	-0.10	0.17	0.04	0.00	0.03	-0.06	1.00							
Maternal involvement decision making	**8**	0.01	-0.11	0.02	-0.04	0.06	-0.01	-0.05	1.00						
Number of older siblings	**9**	0.10	-0.15	-0.08	0.05	-0.02	0.23	-0.18	0.03	1.00					
Household size	**10**	0.05	0.02	-0.08	0.07	-0.02	0.10	-0.01	-0.07	0.20	1.00				
Wealth quintile	**11**	-0.08	0.15	0.15	-0.06	0.02	0.05	0.28	0.05	-0.10	0.06	1.00			
Religion	**12**	-0.03	0.02	-0.01	-0.05	0.01	0.00	0.02	0.05	-0.07	-0.13	0.02	1.00		
Health insurance	**13**	0.05	0.00	-0.05	-0.03	-0.04	0.12	-0.02	0.02	0.06	0.08	0.05	-0.06	1.00	
Self-help group	**14**	0.02	0.00	0.05	-0.02	-0.03	0.05	-0.03	0.04	0.22	-0.01	0.04	0.01	0.08	1.00

The analytical sample includes all respondents with card verification and complete data. Pearson's R correlation statistic with a cutoff of r > 0.5. All listed variables were introduced into the multivariable model.

Table B. Association of perinatal care (birth setting and timely PNC) with the vaccination status of children aged 10-20 months in Bihar. Analytical sample, unadjusted OR.

	**Full vaccination**	**Any vaccination**				
**Predicting variables**	All	BCG	Polio	Hepatitis B	DPT	Measles
**Birth setting** (Ref: Home birth)						
Institutional, public	1.11	1.92	**1.69***	**4.37*****	1.37	1.18
	[0.78,1.57]	[0.60,6.16]	[1.02,2.79]	[2.75,6.96]	[0.91,2.06]	[0.70,1.98]
Institutional, private	1.76	0.74	0.60	1.62	0.84	2.41
	[0.98,3.16]	[0.19,2.82]	[0.26,1.39]	[0.86,3.05]	[0.42,1.67]	[0.64,9.13]
**Extra care**						
Timely PNC	**1.47***	1.64	**3.09*****	**1.99***	**2.08****	1.01
	[1.06,2.05]	[0.73,3.72]	[1.63,5.85]	[1.17,3.39]	[1.24,3.46]	[0.64,1.59]
Observations	809	809	809	809	809	809
Prob > chi^2	0.04	0.16	0.00	0.00	0.01	0.61

Boldface stars indicate statistical significance: * (p<0.05), ** (p<0.01), *** (p<0.001). Logistic regression results are presented as OR with confidence interval in brackets. The analytical sample includes all respondents with card verification and complete data. Odds of having received at least a single vaccination for BCG, polio, hepatitis B or DPT vaccination are compared by birth setting and the provision of timely PNC. Further odds of having received full vaccination for all recommended vaccines (All) are compared by birth setting and the provision of timely PNC. Ref, Reference; OR, Odds ratio; BCG, Bacille Calmette-Guerin; DPT, diphtheria/pertussis/tetanus.

Table C. Association of perinatal care (birth setting and timely PNC) with the vaccination status of children aged 10-20 months in Bihar. Analytical sample including all covariates.

	**Full vaccination**	**Any vaccination**				
**Predicting variables**	All	BCG	Polio	Hepatitis B	DPT	Measles
**Birth setting** (Ref: Home birth)						
Institutional, public	1.07	1.97	1.59	**4.86*****	1.36	1.03
	[0.74,1.55]	[0.69,5.64]	[0.95,2.66]	[2.97,7.95]	[0.89,2.07]	[0.59,1.77]
Institutional, private	1.55	1.05	0.52	1.51	0.78	1.77
	[0.82,2.93]	[0.26,4.23]	[0.20,1.34]	[0.71,3.22]	[0.37,1.64]	[0.41,7.60]
**Extra care**						
Timely PNC	**1.48***	1.93	**3.37*****	**2.11****	**2.29****	0.94
	[1.06,2.08]	[0.82,4.53]	[1.79,6.36]	[1.24,3.59]	[1.35,3.88]	[0.58,1.52]
Received at least one ANC visit	1.18	0.74	1.31	0.88	1.46	**1.73***
	[0.82,1.70]	[0.24,2.27]	[0.75,2.28]	[0.53,1.46]	[0.95,2.22]	[1.03,2.92]
**Child characteristics**						
Sex of child	**1.30***	0.78	1.33	1.04	1.22	1.30
	[1.03,1.63]	[0.33,1.82]	[0.80,2.22]	[0.69,1.57]	[0.85,1.76]	[0.88,1.93]
Age of the child > 12 months	0.84	1.74	1.79	0.22	1.40	1.49
	[0.42,1.68]	[0.25,12.08]	[0.45,7.11]	[0.02,2.28]	[0.40,4.93]	[0.32,6.89]
Number of older siblings	0.90	0.87	0.88	**0.80***	0.91	1.07
	[0.79,1.03]	[0.63,1.19]	[0.68,1.15]	[0.67,0.96]	[0.77,1.08]	[0.88,1.29]
**Maternal characteristics**						
Age *(Ref: < 24 years)*						
24-34 years	1.13	0.95	0.89	1.36	1.29	0.90
	[0.76,1.68]	[0.29,3.12]	[0.46,1.72]	[0.83,2.24]	[0.81,2.07]	[0.53,1.53]
35 years or older	1.51	1.17	1.39	1.77	1.20	1.72
	[0.91,2.52]	[0.29,4.70]	[0.69,2.80]	[0.93,3.40]	[0.66,2.19]	[0.90,3.28]
Education *(Ref: No schooling)*						
Primary school	0.94	1.97	1.40	0.73	0.91	1.35
	[0.52,1.70]	[0.30,12.87]	[0.61,3.19]	[0.31,1.68]	[0.49,1.70]	[0.56,3.24]
Middle school or higher	1.18	0.86	1.55	0.87	1.01	**2.54***
	[0.79,1.74]	[0.32,2.28]	[0.86,2.81]	[0.47,1.64]	[0.64,1.60]	[1.20,5.37]
Maternal involvement health care child	**2.19*****	0.52	1.83	1.55	**1.84***	1.45
	[1.47,3.25]	[0.15,1.74]	[0.99,3.36]	[1.00,2.41]	[1.16,2.93]	[0.83,2.54]
Self-help group	1.52	2.44	1.47	0.92	0.99	1.54
	[0.98,2.34]	[0.72,8.20]	[0.84,2.60]	[0.55,1.54]	[0.65,1.51]	[0.84,2.82]
**Household characteristics**						
Household size	**1.09*****	**1.09***	**1.11***	**1.11*****	**1.10****	0.98
	[1.04,1.15]	[1.01,1.19]	[1.02,1.21]	[1.05,1.17]	[1.02,1.18]	[0.91,1.06]
Health insurance	1.26	0.74	0.97	0.84	1.55	1.19
	[0.95,1.68]	[0.31,1.76]	[0.50,1.90]	[0.51,1.37]	[0.88,2.74]	[0.71,2.00]
Hindu	**1.90***	1.13	0.66	**2.15****	**1.85***	1.61
	[1.05,3.44]	[0.33,3.88]	[0.30,1.46]	[1.27,3.65]	[1.03,3.30]	[0.91,2.85]
Wealth quintile *(Ref: Poorest)*						
Poorer	0.99	0.34	1.17	1.53	0.77	0.88
	[0.62,1.59]	[0.07,1.64]	[0.50,2.73]	[0.71,3.26]	[0.36,1.63]	[0.49,1.60]
Middle	1.17	0.75	0.78	1.78	0.58	0.76
	[0.70,1.94]	[0.18,3.14]	[0.27,2.25]	[0.85,3.70]	[0.25,1.33]	[0.39,1.48]
Richer	1.33	0.65	0.74	1.90	0.60	0.76
	[0.80,2.23]	[0.08,5.25]	[0.29,1.90]	[0.96,3.76]	[0.25,1.44]	[0.39,1.48]
Richest	0.96	0.24	0.45	0.89	**0.39***	0.66
	[0.54,1.69]	[0.04,1.54]	[0.16,1.25]	[0.42,1.88]	[0.16,0.96]	[0.25,1.73]
Observations	809	809	809	809	809	809
Prob > chi^2	0.00	0.00	0.01	0.00	0.00	0.33

Boldface stars indicate statistical significance: * (p<0.05), ** (p<0.01), *** (p<0.001). Logistic regression results are presented as OR with confidence interval in brackets. The analytical sample includes all respondents with card verification and complete data. Standard errors were clustered on panchayat level. Odds of having received at least a single vaccination for BCG, polio, hepatitis B or DPT vaccination are compared by birth setting and the provision of timely PNC. Further odds of having received full vaccination for all recommended vaccines (All) are compared by birth setting and the provision of timely PNC. Ref, Reference; OR, Odds ratio; BCG, Bacille Calmette-Guerin; DPT, diphtheria/pertussis/tetanus.

Table D. Association of perinatal care (birth setting and timely PNC) with the vaccination status of children aged 10-20 months in Bihar. Analytical sample including all covariates and block fixed effects.

	Full vaccination	Any vaccination				
**Predicting variables**	All	BCG	Polio	Hepatitis B	DPT	Measles
**Birth setting** (Ref: Home birth)						
Institutional, public	0.94	1.89	1.67	**4.74*** **	1.42	0.95
	[0.66,1.34]	[0.62,5.76]	[0.97,2.89]	[2.80,8.03]	[0.96,2.09]	[0.55,1.62]
Institutional, private	1.39	1.20	0.55	1.49	0.82	1.65
	[0.71,2.71]	[0.30,4.90]	[0.21,1.44]	[0.68,3.28]	[0.39,1.70]	[0.38,7.15]
**Extra care**						
Timely PNC	**1.42***	1.84	**3.33*** **	**2.07***	**2.23** **	0.91
	[1.00,2.00]	[0.79,4.30]	[1.72,6.45]	[1.19,3.59]	[1.30,3.82]	[0.57,1.46]
Received at least one ANC visit	1.18	0.80	1.32	0.92	1.46	**1.71***
	[0.81,1.72]	[0.26,2.49]	[0.75,2.32]	[0.55,1.53]	[0.94,2.25]	[1.03,2.82]
**Child characteristics**						
Sex of child	**1.29***	0.76	1.39	1.00	1.27	1.26
	[1.02,1.63]	[0.31,1.88]	[0.80,2.40]	[0.64,1.56]	[0.85,1.89]	[0.86,1.86]
Age of the child > 12 months	0.86	2.12	1.75	0.22	1.31	1.85
	[0.42,1.77]	[0.40,11.24]	[0.46,6.71]	[0.02,2.37]	[0.32,5.29]	[0.41,8.44]
Number of older siblings	0.90	0.87	0.88	**0.81***	0.92	1.10
	[0.78,1.04]	[0.62,1.22]	[0.67,1.16]	[0.67,0.98]	[0.76,1.10]	[0.91,1.34]
**Maternal characteristics**						
Age *(Ref: < 24 years)*						
24-34 years	1.12	0.86	0.93	1.41	1.37	0.86
	[0.72,1.74]	[0.22,3.40]	[0.49,1.76]	[0.86,2.33]	[0.88,2.15]	[0.51,1.45]
35 years or older	1.88	1.13	1.52	1.96	1.51	1.54
	[1.00,3.53]	[0.25,5.11]	[0.74,3.10]	[0.97,4.00]	[0.80,2.85]	[0.77,3.09]
Education *(Ref: No schooling)*						
Primary school	0.97	2.10	1.43	0.72	0.96	1.31
	[0.54,1.76]	[0.31,14.16]	[0.64,3.18]	[0.31,1.66]	[0.54,1.73]	[0.54,3.16]
Middle school or higher	1.22	0.88	1.57	0.90	1.07	**2.44***
	[0.83,1.79]	[0.34,2.22]	[0.88,2.78]	[0.50,1.65]	[0.69,1.66]	[1.13,5.25]
Maternal involvement health care child	**2.15*** **	0.48	1.83	1.50	**1.83***	1.41
	[1.45,3.19]	[0.14,1.71]	[0.98,3.42]	[0.96,2.33]	[1.14,2.93]	[0.79,2.52]
Self-help group	1.47	2.25	1.53	0.92	1.02	1.50
	[0.97,2.24]	[0.66,7.68]	[0.87,2.67]	[0.55,1.53]	[0.67,1.56]	[0.83,2.73]
**Household characteristics**						
Household size	**1.08** **	1.04	**1.12***	**1.08***	**1.11***	0.97
	[1.03,1.14]	[0.95,1.14]	[1.02,1.22]	[1.02,1.15]	[1.03,1.21]	[0.90,1.04]
Health insurance	1.23	0.77	0.95	0.84	1.52	1.12
	[0.93,1.64]	[0.31,1.93]	[0.47,1.89]	[0.50,1.39]	[0.86,2.72]	[0.66,1.90]
Hindu	1.62	1.07	0.62	**1.98***	1.69	1.56
	[0.91,2.87]	[0.30,3.87]	[0.27,1.42]	[1.10,3.57]	[0.93,3.05]	[0.91,2.66]
Wealth quintile *(Ref: Poorest)*						
Poorer	1.00	0.40	1.18	1.54	0.77	0.84
	[0.61,1.65]	[0.07,2.20]	[0.50,2.77]	[0.71,3.34]	[0.35,1.66]	[0.46,1.53]
Middle	1.26	0.92	0.73	1.81	0.55	0.77
	[0.75,2.11]	[0.20,4.18]	[0.24,2.15]	[0.87,3.79]	[0.24,1.28]	[0.39,1.50]
Richer	1.36	0.76	0.69	1.87	0.54	0.74
	[0.79,2.34]	[0.09,6.21]	[0.26,1.83]	[0.94,3.70]	[0.22,1.36]	[0.38,1.44]
Richest	1.00	0.30	0.41	0.88	**0.35***	0.66
	[0.55,1.83]	[0.04,2.16]	[0.14,1.24]	[0.40,1.92]	[0.13,0.92]	[0.26,1.73]
Observations	809	744	809	809	809	809
Prob > chi^2	0.00	0.00	0.01	0.00	0.00	0.13

Boldface stars indicate statistical significance: * (p<0.05), ** (p<0.01), *** (p<0.001). Logistic regression results are presented as OR with confidence interval in brackets. The analytical sample includes all respondents with card verification and complete data. Standard errors were clustered on panchayat level. Fixed effects controlling for block characteristics were introduced to the model. Odds of having received at least a single vaccination for BCG, polio, hepatitis B or DPT vaccination are compared by birth setting and the provision of timely PNC. Further odds of having received full vaccination for all recommended vaccines (All) are compared by birth setting and the provision of timely PNC. Ref, Reference; OR, Odds ratio; BCG, Bacille Calmette-Guerin; DPT, diphtheria/pertussis/tetanus.

Table E. Association of perinatal care (birth setting and timely PNC) with the vaccination status of children aged 10-20 months in Bihar. Full sample (card verification and maternal recall), unadjusted OR.

	**Full vaccination**		**Any vaccination**				
**Predicting variables**	All		BCG	Polio	Hepatitis B	DPT	Measles
**Birth setting** (Ref: Home birth)							
Institutional, public	1.14		1.61	1.56	**3.74*****	1.31	1.33
	[0.83,1.57]		[0.72,3.61]	[0.99,2.47]	[2.52,5.55]	[0.90,1.91]	[0.88,2.00]
Institutional, private	1.48		0.58	0.54	1.35	0.70	1.69
	[0.88,2.50]		[0.21,1.57]	[0.25,1.16]	[0.71,2.56]	[0.35,1.41]	[0.62,4.62]
**Extra care**							
Timely PNC	**1.63****		1.76	**3.10*****	**2.29*****	**2.27*****	0.95
	[1.22,2.19]		[0.96,3.21]	[1.71,5.63]	[1.43,3.68]	[1.47,3.51]	[0.64,1.41]
Observations	1085	1083	1079	1078	1071	1063	
Prob > chi^2	0.00	0.02	0.00	0.00	0.00	0.56	

Boldface stars indicate statistical significance: * (p<0.05), ** (p<0.01), *** (p<0.001). Logistic regression results are presented as OR with confidence interval in brackets. The full sample includes all respondents with complete data by card verification and maternal recall. Odds of having received at least a single vaccination for BCG, polio, hepatitis B or DPT vaccination are compared by birth setting and the provision of timely PNC. Further odds of having received full vaccination for all recommended vaccines (All) are compared by birth setting and the provision of timely PNC. Ref, Reference; OR, Odds ratio; BCG, Bacille Calmette-Guerin; DPT, diphtheria/pertussis/tetanus.

Table F. Association of perinatal care (birth setting and timely PNC) with the vaccination status of children aged 10-20 months in Bihar. Full sample (card verification and maternal recall) including all covariates.

Predicting variables	Full vaccination	Any vaccination				
	All	BCG	Polio	Hepatitis B	DPT	Measles
**Birth setting** (Ref: Home birth)						
Institutional, public	1.21	1.51	1.50	**4.30*** **	1.34	1.22
	[0.87,1.70]	[0.73,3.11]	[0.93,2.42]	[2.78,6.65]	[0.91,1.97]	[0.78,1.92]
Institutional, private	1.51	0.69	0.49	1.31	0.70	1.41
	[0.85,2.66]	[0.23,2.07]	[0.21,1.15]	[0.64,2.67]	[0.33,1.48]	[0.51,3.96]
**Extra care**						
Timely PNC	**1.59** **	**2.01***	**2.91*** **	**2.10** **	**2.20** **	0.92
	[1.15,2.21]	[1.06,3.79]	[1.63,5.21]	[1.31,3.36]	[1.37,3.51]	[0.61,1.39]
Received at least one ANC visit	1.05	0.94	1.38	0.94	1.45	1.55
	[0.77,1.45]	[0.40,2.23]	[0.86,2.20]	[0.58,1.53]	[0.96,2.20]	[0.96,2.51]
**Child characteristics**						
Sex of child	1.27	1.11	1.34	1.07	1.24	1.26
	[1.00,1.61]	[0.52,2.39]	[0.83,2.18]	[0.73,1.57]	[0.85,1.80]	[0.87,1.83]
Age of the child > 12 months	1.11	**3.24***	1.69	0.42	1.79	1.27
	[0.62,2.00]	[1.10,9.49]	[0.60,4.76]	[0.10,1.75]	[0.73,4.35]	[0.40,4.01]
Number of older siblings	0.93	1.08	0.89	0.86	0.93	1.08
	[0.81,1.05]	[0.85,1.37]	[0.70,1.14]	[0.72,1.02]	[0.79,1.11]	[0.90,1.29]
Card verification	**2.17*** **	**0.37** **	**3.09***	**4.82*** **	**3.64*** **	0.92
	[1.42,3.33]	[0.18,0.78]	[1.29,7.36]	[2.27,10.27]	[1.84,7.21]	[0.59,1.45]
**Maternal characteristics**						
Age *(Ref: < 24 years)*						
24-34 years	1.17	1.01	0.86	1.30	1.20	0.89
	[0.83,1.65]	[0.44,2.30]	[0.46,1.63]	[0.83,2.04]	[0.77,1.88]	[0.55,1.47]
35 years or older	1.33	0.68	1.07	1.42	1.06	1.57
	[0.81,2.17]	[0.28,1.66]	[0.54,2.09]	[0.81,2.49]	[0.59,1.89]	[0.86,2.88]
Education *(Ref: No schooling)*						
Primary school	0.80	0.62	1.13	0.70	0.88	0.93
	[0.48,1.32]	[0.22,1.73]	[0.48,2.64]	[0.33,1.47]	[0.49,1.57]	[0.48,1.81]
Middle school or higher	1.21	0.97	1.48	0.88	1.07	1.88
	[0.84,1.74]	[0.43,2.21]	[0.78,2.80]	[0.50,1.54]	[0.68,1.69]	[0.97,3.66]
Maternal involvement health care child	**2.40*** **	1.02	1.73	**1.52***	**1.84** **	**1.77** **
	[1.77,3.26]	[0.53,1.98]	[0.98,3.06]	[1.01,2.29]	[1.23,2.75]	[1.15,2.71]
Self-help group	1.35	1.50	1.28	0.92	1.01	1.33
	[0.95,1.92]	[0.60,3.76]	[0.77,2.13]	[0.57,1.50]	[0.68,1.49]	[0.80,2.20]
**Household characteristics**						
Household size	**1.09*** **	0.98	1.07	**1.06***	**1.07***	0.98
	[1.04,1.14]	[0.90,1.06]	[0.98,1.16]	[1.00,1.13]	[1.01,1.14]	[0.93,1.04]
Health insurance	1.18	0.84	0.81	0.78	1.36	1.04
	[0.90,1.53]	[0.40,1.78]	[0.44,1.48]	[0.51,1.18]	[0.82,2.25]	[0.65,1.68]
Hindu	**1.78***	0.80	0.73	**1.92***	1.61	1.49
	[1.11,2.86]	[0.26,2.47]	[0.37,1.44]	[1.11,3.33]	[0.92,2.80]	[0.87,2.55]
Wealth quintile *(Ref: Poorest)*						
Poorer	0.93	0.48	1.08	1.45	0.77	0.89
	[0.58,1.50]	[0.15,1.57]	[0.48,2.43]	[0.70,2.99]	[0.37,1.60]	[0.49,1.62]
Middle	1.03	0.57	0.62	1.33	0.52	0.63
	[0.63,1.69]	[0.20,1.60]	[0.24,1.65]	[0.69,2.56]	[0.25,1.06]	[0.36,1.11]
Richer	1.23	1.09	0.81	**1.94***	0.63	0.63
	[0.77,1.95]	[0.23,5.13]	[0.34,1.94]	[1.02,3.69]	[0.29,1.40]	[0.34,1.16]
Richest	0.91	0.47	0.47	0.97	**0.42***	0.63
	[0.55,1.51]	[0.14,1.58]	[0.19,1.18]	[0.50,1.88]	[0.19,0.92]	[0.29,1.34]
Observations	1085	1083	1079	1078	1071	1063
Prob > chi^2	0.00	0.00	0.00	0.00	0.00	0.01

Boldface stars indicate statistical significance: * (p<0.05), ** (p<0.01), *** (p<0.001). Logistic regression results are presented as OR with confidence interval in brackets. The full sample includes all respondents with complete data by card verification and maternal recall. Standard errors were clustered on panchayat level. Odds of having received at least a single vaccination for BCG, polio, hepatitis B or DPT vaccination are compared by birth setting and the provision of timely PNC. Further odds of having received full vaccination for all recommended vaccines (All) are compared by birth setting and the provision of timely PNC. Ref, Reference; OR, Odds ratio; BCG, Bacille Calmette-Guerin; DPT, diphtheria/pertussis/tetanus.

Table G. Association of perinatal care (birth setting and timely PNC) with the vaccination status of children aged 10-20 months in Bihar. Full sample (card verification and maternal recall) including all covariates and block fixed effects.

	Full vaccination	Any vaccination				
**Predicting variables**	All	BCG	Polio	Hepatitis B	DPT	Measles
**Birth setting** (Ref: Home birth)						
Institutional, public	1.14	1.57	1.62	**4.24*** **	1.39	1.19
	[0.81,1.59]	[0.73,3.38]	[0.99,2.63]	[2.69,6.68]	[0.98,1.98]	[0.77,1.83]
Institutional, private	1.41	0.65	0.53	1.29	0.72	1.39
	[0.78,2.55]	[0.21,1.99]	[0.22,1.28]	[0.61,2.76]	[0.34,1.51]	[0.50,3.90]
**Extra care**						
Timely PNC	**1.51***	**1.96***	**2.88*** **	**2.03** **	**2.16** **	0.90
	[1.08,2.11]	[1.03,3.75]	[1.58,5.23]	[1.25,3.30]	[1.34,3.48]	[0.60,1.34]
Received at least one ANC visit	1.06	1.07	1.42	0.99	1.49	1.55
	[0.76,1.47]	[0.43,2.63]	[0.89,2.27]	[0.60,1.61]	[0.98,2.26]	[0.96,2.51]
Observations	1085	988	1079	1078	1071	1063
Prob > chi^2	0.00	0.00	0.01	0.00	0.00	0.01

Boldface stars indicate statistical significance: * (p<0.05), ** (p<0.01), *** (p<0.001). Logistic regression results are presented as OR with confidence interval in brackets. The full sample includes all respondents with complete data by card verification and maternal recall. Standard errors were clustered on panchayat level. Fixed effects controlling for block characteristics were introduced to the model. Odds of having received at least a single vaccination for BCG, polio, hepatitis B or DPT vaccination are compared by birth setting and the provision of timely PNC. Further odds of having received full vaccination for all recommended vaccines (All) are compared by birth setting and the provision of timely PNC. Ref, Reference; OR, Odds ratio; BCG, Bacille Calmette-Guerin; DPT, diphtheria/pertussis/tetanus.

Table H. Number of missing values (n and %) for each variable.

Missings	N	%
Sample size	1967	
Households revisited	1612	
Vaccination evidence	1442	100
Card verification	1011	
Birth setting	5	0.49
ANC	15	1.48
PNC	52	5.14
Sex of the child	0	0.00
Age of the child	0	0.00
Age of mother	20	1.98
Education mother	15	1.48
Health decision mother	2	0.20
Number of older siblings	54	5.34
Wealth quintile	54	5.34
Religion	10	0.99
Insurance	2	0.20
Household size	0	0.00
Self-help-group	12	1.19
Full_all	0	0.00
Any_BCG	0	0.00
Any_Polio	0	0.00
Any_HepB	0	0.00
Any_DPT	0	0.00
Any_measles	0	0.00
Analytical sample	809	

1442 participants were able to provide information about their vaccination evidence and were thus considered eligible for the main analysis.
